# Application of TD-DFT Theory to Studying Porphyrinoid-Based Photosensitizers for Photodynamic Therapy: A Review

**DOI:** 10.3390/molecules26237176

**Published:** 2021-11-26

**Authors:** Agnieszka Drzewiecka-Matuszek, Dorota Rutkowska-Zbik

**Affiliations:** Jerzy Haber Institute of Catalysis and Surface Chemistry Polish Academy of Sciences, Niezapominajek 8, 30-239 Krakow, Poland; agnieszka.drzewiecka-matuszek@ikifp.edu.pl

**Keywords:** porphyrinoids, photodynamic therapy (PDT), time-dependent density functional theory (TD-DFT)

## Abstract

An important focus for innovation in photodynamic therapy (PDT) is theoretical investigations. They employ mostly methods based on Time-Dependent Density Functional Theory (TD-DFT) to study the photochemical properties of photosensitizers. In the current article we review the existing state-of-the-art TD-DFT methods (and beyond) which are employed to study the properties of porphyrinoid-based systems. The review is organized in such a way that each paragraph is devoted to a separate aspect of the PDT mechanism, e.g., correct prediction of the absorption spectra, determination of the singlet–triplet intersystem crossing, and interaction with molecular oxygen. Aspects of the calculation schemes are discussed, such as the choice of the most suitable functional and inclusion of a solvent. Finally, quantitative structure–activity relationship (QSAR) methods used to explore the photochemistry of porphyrinoid-based systems are discussed.

## 1. Introduction

**Scheme 1:**
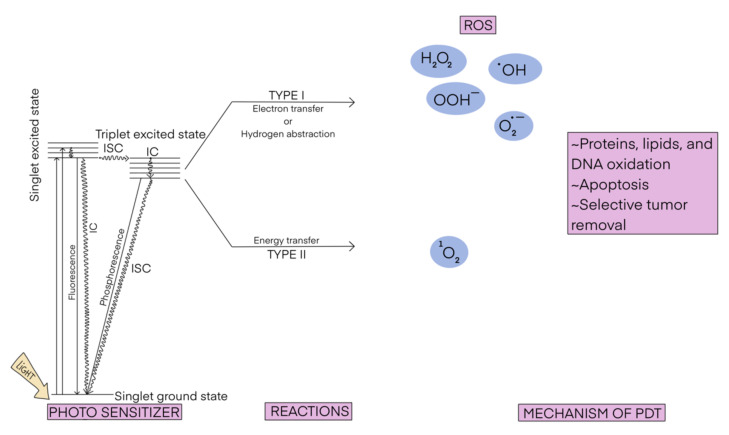
The scheme of photodynamic therapy.

Photodynamic therapy (PDT) is a possible treatment for various diseases [1,2,3], such as different kinds of cancer [4,5,6]; bacterial [7,8], fungal [9], and viral infections (including Covid-19) [10,11]; cutaneous manifestations [12]; dental caries [13]; and rheumatoid arthritis [14]. The mechanism of the PDT is schematically displayed in Scheme 1. It consists of the irradiation of infected/malignant cells in the presence of a light-absorbing compound (called photosensitizer). The photosensitizer is excited from its ground state (usually the singlet state S_0_) to the excited state (usually the first excited singlet state S_1_), which then undergoes intersystem crossing (and a series of de-excitations), generating an excited triplet state (T_1_). T_1_ is then deactivated to S_0_ following one of two processes. In the typical scenario, known also as type II photoreaction, the photosensitizer transfers its energy to the O_2_ molecule yielding singlet oxygen ^1^O_2_. In the less common process, known as type I photoreaction, the photosensitizer in the T_1_ state reacts with a molecule present nearby (e.g., from the cell membrane) and converts it into a highly reactive species (e.g., NO, O_2_^−^, ROO, or RO). Both processes can occur simultaneously and the ratio between them depends on the type of photosensitizer and the physico-chemical conditions in the place of action, mostly the concentration of oxygen. No matter which type of photoreaction occurs, the emerging reactive species are able to damage the targeted cells.

In PDT, typically one uses visible light, which is able to penetrate tissues in the so-called therapeutic window to centimeters of depth [15], thereby reaching shallow tumors only. In order to overcome this limitation and target deeper tumors, other light sources have been tested [16]. Among them, electromagnetic radiation such as near infrared light [16,17,18], Cherenkov radiation [19,20,21], and X-rays [22,23,24,25] have been used. Self-illuminated systems based on chemiluminescent and/or bioluminescent mechanisms [16,26,27,28] are also under study.

Common photosensitizers originate usually from the following classes of molecules: different forms of porphyrins, including benzoporphyrins, hexaphyrins, and sapphyrins; porphyrin precursors—aminolevulinic acid (ALA); chlorins; bacteriochlorins; phthalocyanines; naphtalocyanines; corroles; indocyanine dyes; BODIPY dyes; Ru (II) complexes; Ir(III) complexes; Au (III) complexes; metal–organic frameworks (MOFs); covalent organic frameworks (COFs); hydrogen-bonded organic frameworks (HOFs); polymers; carbon-based nanomaterials; silicon-based nanomaterials; pure metals; transition metal carbides (TMCs); TiO_2_; mono- and bimetal oxides; and bimetal sulfides [4,29,30,31,32,33]. Among them, porphyrinoids have been in use the longest, are the most studied, and are the most widely known. Most of photosensitizers accepted for clinical use come from this group [1]. Additionally, their structure allows various modifications, such as metalation or substitution of the porphyrin ring, to improve their photophysical properties or to construct biohybrid materials [34]. In this context, porphyrinoids may also have dual effects as therapeutic and diagnostic agents, e.g., in PET imaging with ^64^Cu or in MRI with Gd as a central atom [31]. Their photodynamic actions are usually exerted via type II photoreactions, i.e., they transfer the excitation energy from the triplet excited state to the oxygen molecule. 

The mechanism of PDT enforces the following key requirements of effective photosensitizers: (1) the ability to absorb light to the first excited singlet state in the so-called therapeutic window (600–800 nm); (2) a high intersystem spin-crossing probability, enabling effective population of the triplet excited state; (3) a T_1_–S_0_ energy gap larger than 0.98 eV, which is the amount of energy required to activate molecular oxygen.

Nowadays, new studies on photosensitizers, in particular, on their properties and activity, are based not only on experimental work, but are also complemented by theoretical calculations. They allow for determination of the geometries and electronic parameters of photosensitizers (bond lengths and orders, angles between atoms, charges accumulated on different parts of molecules, polarizabilities, etc.), and their spectroscopic properties (excited state energies, including the T_1_–S_0_ energy gap and estimation of excited state lifetimes). They employ mostly methods based on Time-Dependent Density Functional Theory (TD-DFT), but other, “higher” level theories are also gaining attention due to constantly increasing computational resources.

Time-Dependent Density Functional Theory (TD-DFT) is an extension of Density Functional Theory (DFT) to the time-dependent domain. As proposed by Runge and Gross [35], the central theorem of TD-DFT states that there is a one-to-one correspondence between the external time-dependent potential and the electronic one-body density for any many-body system evolving from a fixed initial state ( Ψ(0)). The time-dependent Schrödinger equation can be solved, and so all observables of the many-electron system, beginning in the initial Ψ(0) state, may be extracted from the one-body time-dependent density. Such an approach is analogous to the Hohenberg–Kohn theorem for the ground-state DFT [36]. extensive descriptions of the TD-DFT methodology can be found in, e.g., [37,38,39]. In 1995, Mark Casida proposed a constructive linear-response formalism for TD-DFT (known as random-phase approximation, RPA), allowing to determine the solutions of the TD-DFT equations [40].

TD-DFT is formally an exact theory, but the implementations require the selection of the exchange correlation functionals. Consequently, the accuracies of the TD-DFT solutions suffer from the limitations of the functionals applied. For instance, in spectroscopic applications, the quality of a solution depends on the system and type of excitation considered. Typical functionals often fail to correctly describe Rydberg and charge-transfer states [41,42]. This can be overcome by the application of range-separated hybrid functionals (see Section 2.2.1 for further discussion). They incorporate a considerable amount of the exact Hartree–Fock exchange at large electron–electron distances and so reflect the correct asymptotic exchange potential.

Despite the limitations outlined above, TD-DFT theory has been used successfully many times to compute properties of various chemical, biological, and physical systems. The most common use is in spectroscopy, where TD-DFT yields response properties and excitations of atoms, molecules, and solids. It also provides the baseline for simulations of real-time dynamics in non-perturbative fields. The success of the TD-DFT approach is due to its relatively high accuracy achieved with relatively low computational cost and ongoing efforts to push its limits. As such, one should mention capturing potential energy surfaces near a conical intersection or addressing the Coulomb blockade phenomenon in calculations of molecular transport [39].

In the current article, we investigate which of the widely available methods seems best suited to studying the physico-chemical properties of porphyrin-based photosensitizers, which are the most common approaches, and which of the descriptors can be predicted with modern theoretical tools.

## 2. Results

The discussion of the literature is divided according to the requirements imposed on the most effective photosensitizers. Each paragraph in a subsection is devoted to a stage of the mechanism of the PDT, e.g., the correct prediction of the photosensitizer structure and its absorption spectra, the determination of singlet–triplet intersystem crossing, and interaction with molecular oxygen. The practical aspects of the calculation schemes are also discussed. These include the choice of the most suitable functional and inclusion of a solvent. Finally, quantitative structure–activity relationship (QSAR) methods to explore the photochemistry of porphyrinoid-based systems are discussed.

### 2.1. Prediction of the Geometric Parameters of Porphyrinoid-Based Photosensitizers

Theoretical calculations of porphyrinoid-based systems are usually done with Density Functional Theory (DFT)—a choice justified by the size of these systems and earlier general benchmark studies [43,44]. The most common is the use of the B3LYP functional [45,46,47,48,49], which is applied to predict geometries of the ground and excited states of the sensitizers [50,51,52], but other functionals, such as M06 [53], are gaining popularity as well [54].

In order to assess the applicability of various computational schemes, the DFT geometries are compared both with the X-ray diffraction (XRD) structures and with those predicted by higher level calculations, because the experimentally derived data can be influenced by the fact that porphyrinoids easily aggregate due to π-stacking.

Typically, B3LYP bond lengths differ by not more than 0.01–0.02 Å as compared to XRD results (B3LYP/6-31G* data for *meso*-tetraphenyl substituted porphyrin (TPP) and its hetero-atom analogs [55] or substituted bacteriochlorins [56]). Much larger discrepancies (up to ca. 20 degrees) have been found for valence angles [56]. The choice of the basis set has only a minor effect on the computed geometries [57]. A study on methyl pheophorbide-*a* revealed, however, that M06-2X/6-311+G(d,p) [53] and LSDA/6-311+G(d,p) [45,47,58] are better than B3LYP at reproducing bond lengths and valence angles, respectively [59].

There are more discrepancies when metal ions are present in the porphyrinoid structure. For instance, a comparison of the B3LYP/SVP geometries of a series of acene-modified zinc porphyrins with those calculated with BH&HLYP/SVP [48,49,60] and ωB97X-D/SVP [61] revealed that the changes in parameters could be up to 0.4 Å, but eventually the electronic parameters were similar and not very geometry-dependent for the neutral, oxidized, and reduced ground state species [50]. Rydberg and Olsen claimed that for iron-containing porphyrins, B3LYP yields the worst results, and that TPSSH [62], PBE0 [63,64], and TPSS [65,66] functionals perform the best (absolute bond distance deviations of 0.015−0.016 Å) [67]. One should be aware, however, that iron-porphyrins are not popular in PDT studies.

Excited state geometries are rarely computed, because they involve long computational times, and comparisons with other theoretical or experimental data are challenging. More is known about the applicability of TD-DFT for obtaining geometries of model systems. Jun Wang and Bo Durbeej studied the applicability of TD-DFT for predicting the excited state geometry of medium-sized organic molecules, including heterocycles [68]. They found that that the studied hybrid functionals (B3LYP, PBE0, M06-2X, CAM-B3LYP [69], and ωB97XD) can reproduce the approximate coupled-cluster singles’ and doubles’ (CC2) excited-state bond lengths with an overall root mean square deviation (RMSD) of 0.011 Å. The BP96 functional [49,70] yielded much worse results. Out of the studied hybrids, B3LYP and PBE0 performed the best. These results were corroborated by the recent findings by Robin Grotjahn and Martin Kaupp, who reported that local-hybrid functionals reproduce better C-N, C-C, C-halogen, and element-H bond lengths in the excited state than other semi-local and range-separated hybrids (the calculations were done for a set of small molecules, and the data were compared with coupled cluster results) [71]. Bond angles are system-dependent, and it was not possible to draw general conclusions for the studied functionals. 

Winter and co-workers determined the first excited state geometries of tetraphenylporphyrin (TPP) and Zn-TPP with B3LYP/TZVP [72]. The appropriateness of the method was then judged based on the comparison of the energy of the first excitation of the resulting structure with experimental results, and/or results calculated with higher-order methods: algebraic diagrammatic construction through second order (ADC(2)), CC2, scaled opposite-spin CC2 (SOS-CC2), and spin-component scaled CC2 (SCS-CC2) within the TZVP basis set. B3LYP was proven to yield reasonable results.

### 2.2. Prediction of the Absorption Spectra

**Scheme 2:**
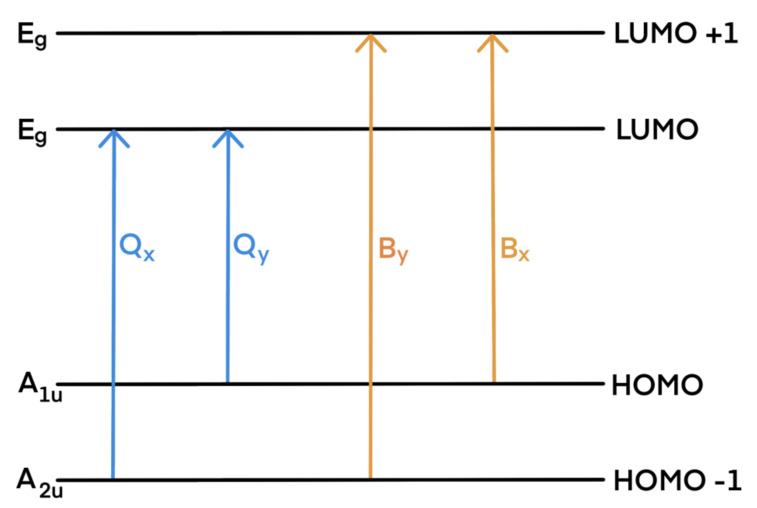
Gouterman’s model of porphyrinoids’ main absorption bands (note that substitution or other modifications in the porphyrin skeleton may result in modification of orbital levels, causing changes in the band assignment).

The experimental absorption spectra of porphyrinoids are divided into two characteristic spectral regions: an intense B band (called also Soret band) located near 350–400 nm (3–3.5 eV) and a weak Q band consisting of four bands in the region 500–800 nm (1.5–2.5 eV). Their origins are usually explained by Gouterman’s four-orbital model [73]. The bands are due to transitions within the two highest occupied molecular orbitals (HOMO, HOMO-1) and the two lowest unoccupied molecular orbitals (LUMO, LUMO-1)—see Scheme 2. The Q and B transitions are x-/y-polarized; therefore, they are denoted as Qx and Qy, and Bx and By, respectively.

Theoretical calculations of the excited states of porphyrinoids are done with Time-Dependent Density Functional Theory or with higher level ab initio methods.

Literature concerning the applicability of the TD-DFT method for predicting adsorption spectra is very rich, and a number of excellent reviews exist—see, e.g., [74,75,76,77,78,79,80,81]. Those reviews indicated that there is no universal functional able to correctly describe all classes of dyes. In case of porphyrinoids, which are at focus of our study, it has been shown that there are two parameters which influence the results the most. These are (i) the choice of functional and (ii) the inclusion of a solvent. Therefore, in the following, those two aspects are presented. The choice of the basis set does not influence the results [82,83].

#### 2.2.1. The Choice of Functional

A TD-DFT functional for UV-VIS spectra simulation can be benchmarked not only against experimental data (ultraviolet-visible light (UV-VIS) or magnetic circular dichroism (MCD) spectroscopy) [84,85], but also using high-level ab initio calculations. The latter option is of particular use when new, to-be-synthetized compounds are proposed for PDT.

Among the plethora of functionals, the most often used to simulate the spectra of porphyrins are: B3LYP, CAM-B3LYP, and PBE0 [51,83,86,87,88,89]. Those choices are well justified in light of the extended study presented recently by Batra and co-workers [83]. They found that hybrid functionals reproduce experimental porphyrinoid spectra with considerably smaller error rates than local general gradient functionals—see Table 1 for a comparison of the mean errors, the mean absolute errors, and the absolute maximum errors determined by comparing computed spectra of various porphyrinoids with different functionals to the experimental values. 

The authors recommend energy scaling procedure for general hybrid functionals, whereas no scaling is required for range-separated hybrid ones. This is in agreement with the previous studies on the applicability of different functionals (including long-range separated and meta hybrid ones) to reproducing the absorption spectra of unsubstituted porphyrin [90] and Tookad® (Pd-bacteriopheophorbide) [82].

Comparisons between the theoretical and experimental spectra are often performed. Based on numerous reports, it is usually advised to use CAM-B3LYP to predict adsorption spectra [86]; see Table 2. The discrepancies between the energies of the theoretical and experimental Q and B bands are usually ca. 0.2 and 0.4 eV, respectively (calculations for porphyrins at CAM-B3LYP/6-31G and CAM-B3LYP/6-31G*—theoretical excitation energies overestimate the experimental ones) [51]. Analogous errors regarding the use of B3LYP for computing the UV–VIS spectra of free-base porphyrins were found to amount to 0.3 and 0.2 eV, respectively [51]; see Table 3. Additionally, B3LYP is sometimes reported to significantly underestimate the oscillator strength [91]. PBE0/6-31+G(d) results in a discrepancy of 0.4 eV for both bands [87]. M05-2X results in 0.3 and 0.5 eV errors for Q and B bands, respectively [92].

It is sometimes claimed that B3LYP and PBE0 reverse the energy ordering between the B and N (5^1^A and 6^1^A) transitions [96].

The success of CAM-B3LYP (and other range-separated functionals) is due to the fact that it includes a long-range correction of the exchange potential, which incorporates an increasing fraction of Hartree–Fock (HF) exchange as the interelectronic separation increases.

#### 2.2.2. Inclusion of Solvent 

In theoretical calculations, solvent can be modelled by inclusion of the solvent molecules into the theoretical model (the so-called explicit solvation scheme) or by immersing the studied molecule inside a cavity in a continuum of a given dielectric constant (the so-called implicit solvation scheme). Most of the DFT calculations for PDT use the second approach. The most popular is the polarized continuum model (PCM), which has several variants, such as dielectric D-PCM and the conductor-like polarized continuum model (C-PCM), also called COSMO [97,98]—see, e.g., [87,99,100].

A comparison of the PCM and C-PCM results for Zn-TPP and its analogues revealed that both approaches yield similar results, which agrees with the experimental values. A slight decrease in computational time for C-PCM as compared with PCM was found [101]. 

Further, inclusion of a solvent within TD-DFT can be accomplished either via linear response (LR) method or external iteration (EI) (known also as the state-specific method, SS). Whereas in the first case, the excitation energies are determined without computing the exact excited state electronic density, in the latter, a different Schrödinger equation is solved for each excited state [102,103,104]. These approaches were tested by M. Dulski and coworkers [89], who computed the geometries and photochemical properties of two derivatives of *meso*-tetraphenylporphyrin (*meso*-tetrakis (3-methoxy-4-hydroxyphenyl)-porphyrin and *meso*-tetrakis (3,5-dimethoxy-4-hydroxyphenyl)-porphyrin) in acetonitrile. Both calculation schemes yielded spectra in agreement with experimental data; however, a small red shift of the spectrum resulted from the LR approach as compared with the EI one. LR calculations give higher oscillator strengths for both the B and Q bands. The value of the T_1_–S_0_ energy gap remains about the same regardless of the calculation scheme. A comparison of theoretical fluorescence emission spectra with experimental spectra indicated that the LR method gives better results than the EI approach. The LR method produces high values of dipole force; the values produced by EI approximations are significantly lower. This suggests that the effect of the solvent on molecules in excited states has a greater impact on calculations done using the LR method.

Similar results were also obtained by Fukuda et al. [105]. Their results indicate that the LR approach reproduced the experimentally observed trends of the solvatochromic shifts of porphyrin in dichloromethane and benzene (separately), as opposed to EI.

Petit et al. [87] compared results obtained with explicit and implicit solvation schemes (C-PCM) for a set of porphyrinoids (porphyrin, chlorin, bacteriochlorin, pheophytin *a*, porphyrazin, and texaphyrin). The explicit solvation (i.e., inclusion of two or four water molecules for one sensitizer molecule) did not affect the geometry of the studied porphyrinoids, but Q_x_ and B bands were shifted upwards, minimizing the discrepancies between theoretical and experimental data. The C-PCM strongly stabilizes orbitals and makes transition moments higher. As a result, the computed excitations are stronger.

Fukuda et al. also compared results for porphyrin in CCl_2_H_2_ and benzene with explicit and implicit solvation [105]. The calculations reproduced the bathochromic shifts of the B band states.

Peng-Dong Fan and collaborators studied the inclusion of solvent by quantum mechanics/molecular mechanics (QM/MM) calculations of ZnP (being the quantum region) immersed in a 30 Å cubic box with 869 water molecules (being the molecular mechanics region) [106]. The ZnP moiety exhibited small out-of-plane distortion after QM/MM optimization (QM: DFT-B3LYP/VTZ basis set on Zn and 6-31*G for the rest of atoms; MM: SPC/E water model). The values of the vertical excitation energies (VEE) of ZnP in the gas phase and in solution were comparable for both methods. The difference between VEE in gas and in solvent was smaller than 0.02 eV for all states corresponding to the Q band (2^1^A and 3^1^A states) or B band (4^1^A and 5^1^A states) for both the TD-DFT (B3LYP functional, Ahlrich’s VTZ basis set on Zn, and 6-31G* basis set for the rest of the atoms) and the coupled cluster with singles and doubles, with the equation of motion approach (EOMCCSD) methods. The discrepancy was larger for the configuration interaction (CI), but it did not exceed 0.05 eV. When higher excitations were computed, the difference between VEE in solution and in the gas phase was maintained for the wave function methods, but for the TD-DFT it amounted to ca. 0.29 eV. Furthermore, TD-DFT reversed the ordering of the 6^1^A and 7^1^A states compared to the B band. The effect was ascribed to the fact that the 6^1^A and 7^1^A states are of charge transfer character.

Regarding the reproduction of the lowest T_1_ excitation energies of porphyrin, chlorin, and porphyrazine, it was found that B3LYP/6-31G(d,p) calculations within the PCM approach (in benzene and dichloromethane) did not differ considerably in T_1_ excitation energies determined in the gas phase, and well reproduced experimental ones [107].

In our opinion, solvent inclusion should be recommended for the calculations of the properties of the photosensitizers. The requirement of photodynamic therapy is to inject photosensitizers into tissues where the environment differs from the in vitro experimental conditions. Protein and/or lipid matrices inside tissues can affect the geometries of the molecules via interactions with charged groups, electron rich aromatic systems, or H-donors in proximity of the photosensitizer species. Consequently, a molecule’s geometry can be distorted from that encountered in the gas phase. Examples include the formation of salt bridges, the emergence of non-bonding solute–solvent interactions, and changes in the photosensitizer’s protonation state. Theoretical studies have revealed that these environmental factors can considerably affect the spectral properties of molecules (in particular, when charge transfer states are considered) and should not be neglected—see e.g., [108,109,110,111,112,113,114,115]. Therefore, the inclusion of solvent effects into calculations is forecast to be one of hot topics in the years to come.

#### 2.2.3. Prediction of Multiphoton Adsorption Spectra

It is often claimed that one of the major limitations of porphyrins use in PDT is the need to activate them by blue or green light, which penetrates poorly into tissues. This can be overcome by use of two-photon absorption (TPA)—a nonlinear optical process involving the simultaneous absorption of two near-infrared photons. Such an approach allows for high spatial resolution of the PDT event and enables one to target tumors at greater depths. The theoretical studies of two-photon absorption are scarce, but gaining importance and predictive power [116]. The studies are focused on the prediction of the adsorption spectra by calculating two-photon absorption cross-sections or two-photon probabilities, which require quadratic response theory [117,118,119]. The probabilities of the two-photon absorption can be computed either by sum-over-state (SOS) expression or from the single residue of the quadratic response (SRQR) in TD-DFT [120,121]. 

Day et al. showed the sensitivity of TPA cross-section predictions to the accuracy of the functionals used to predict the most relevant state energies. They demonstrated that the overestimation by 0.5 eV (in respect to the experimental data) of the transition energy for the relevant TPA state in [5,15-bis(3,5-bitert-butylphenyl)-10,20-bis(trihexylsilylethynyl)porphyrinato]zinc by B3LYP led to a 5-fold overprediction of the TPA cross-section for this molecule, whereas for 5,5′-(ethyne)bis[[10,20-bis(3,5-bitert- butylphenyl)-porphyrinato]zinc], underestimation of the transition energy by 0.5 eV with CAM-B3LYP and by 0.8 eV with B3LYP lead to underestimations of the TPA cross-section by factors of 2 and 4, respectively [122].

Greco and co-workers computed two-photon absorptivities using the B3LYP/6-31G(d) equilibrium geometries and found that they were very sensitive to the ground-state geometry [123].

Further, it was found that the B3LYP/6-31G* and CAM-B3LYP/6-31G*-computed two-photon cross-sections of porphyrin derivatives with asymmetric substitution at the *meso*-positions were overestimated in comparison with the experimental data [124]. It was shown that all excitation wavelengths were lowered when CAM-B3LYP was used (in contrast to B3LYP results). CAM-B3LYP cross-section values were much smaller than those determined with B3LYP.

Only recently was the possibility of three-photon absorption of porphyrinoids explored experimentally and theoretically [125,126,127]. The results based on the SOS formulas indicate fair agreement between the computational and experimental results for a range of substituted porphyrins. The predicted cross-sections were overestimated by a factor of 2, but all trends were reproduced correctly [125].

### 2.3. Determination of the Singlet–Triplet Intersystem Crossing

The transfer of the excitation energy from the photosensitizer to, e.g., molecular oxygen requires the excited photosensitizer to change its spin state from singlet to triplet. This is done via intersystem crossing. The efficiency of the intersystem spin crossing (ISC) depends on the amplitudes of the spin-orbit matrix elements for the S_1_→T_1_ radiationless transitions, which can be computed with the response theory [128].

The methodology usually employed for spin-orbit coupling, within both variational and perturbative approaches, is presented in, e.g., [129,130].

The rate constant ($k_{S_{i}T_{j}\;}$) for the ISC between the excited *S_i_* state and the *j*th triplet state (*T_j_*) can be estimated as [131]:

$$k_{S_{i}T_{j}\;}=10^{10}{\left|\langle \psi \left(S_{i}\right)\left|{\hat{H}}_{SO}\right|\psi \left(T_{j}\right)\rangle \right|}^{2}\rho$$

where $\langle \psi \left(S_{i}\right)\left|{\hat{H}}_{SO}\right|\psi \left(T_{j}\right)\rangle$ is the matrix element of the spin-orbit coupling interaction operator (expressed here in cm^−1^). For macrocyclic molecules such as porphyrinoids, the main contribution to the Franck–Condon factor arises from vibrational states with energies of ca. 1400 cm^−1^, corresponding to Huang–Rhys factors *y* of ca. 0.3. The density of the final states can then be estimated from the semiempirical expression for the density of states:

$$\rho =\frac{y^{n}e^{-y}}{n!}$$

$$\mathrm{with}n\approx \frac{E\left(S_{i}\right)-E\left(T_{j}\right)}{1400}.$$

The reported calculations employ mostly the B3LYP functional with cc-pVDZ or 6-31G(d) basis sets for substituted TPP and ZnTPP, free and metalated *meso*-substituted tetrabenzotriazaporphyrin, expanded bacteriochlorins, and temocene (the porphycene analogue of Foscan®) [56,95,132,133]. The computed spin-orbit couplings are in line with the trend of intersystem crossing yields determined experimentally.

Alberto and co-workers reported spin-orbit couplings determined for Foscan® and temocene at the B3LYP/6-31G(d) level, showing correlations with the experimental singlet oxygen quantum yields [95]. By contrast, based on the values spin-orbit matrix elements computed for free tetrabenzotriazaporphyrin and its Mg and Zn complexes, the same group explained that these photosensitizers would not be able to act as singlet oxygen activators [133].

De Simone et al. computed the spin-orbit couplings for a set of tetraphenyl porphyrins and their zinc(II) complexes; halogen atoms (F, Cl, Br, I) were introduced into their macrocycle skeletons [132]. It was shown that the computed values of the spin-orbit couplings increased with the halogen atom mass, showing a heavy atom effect. In such a way, an efficient intersystem crossing for different decay routes was ensured. The authors demonstrated that the presence of halogen atoms influenced the composition of relevant orbitals, especially in the triplet state, for which the contribution of the halogen atom lone pairs increased with the atomic mass of the halogens. Based on spin-orbit couplings and S_0_–T_1_ energy gap values, different deactivation channels were considered, out of which the S_1_−T_1_ ISC process was the most probable.

These observations prove that the adopted scheme of calculations, although not frequently used so far, can be used to predict the photochemical properties of the new systems.

### 2.4. Interaction with Molecular Oxygen

Although this step is of crucial importance for PDT, theoretical studies on it are very limited. There are two approaches to tackle the problem of the interaction between the photosensitizer and molecular oxygen.

The first one comprises the comparison of the photosensitizer’s S_0_–T_1_ energy gap with the energy needed to excite oxygen from ^3^O_2_ to ^1^O_2_. The energy needed for the formation of singlet oxygen is 0.98 eV, but experimental studies determined that at least 1.13 eV is needed in solution [134,135,136]. In calculations, one is better to refer to the energy required to excite the triplet molecular oxygen determined at the same level of theory, rather than that used for porphyrinoid characterization (e.g., for the B3LYP/6-31G* it is 0.91 eV [56]).

The theoretical studies of various porphyrinoids done in Russo’s group indicate that the PBE0PBE and B3LYP functionals are the most suitable for reproducing the singlet–triplet energy gaps, and both have an average error of 0.1 eV [137]. B3LYP with both 6-31G and 6-31G(d,p) gives S_0_–T_1_ energy gaps in agreement with experimental results for porphyrin, chlorin, porphyrazine, and zinc porphyrins bearing *meso*-4-methylthiophenium groups [107,138].

The second strategy aims at the theoretical description of the energy transfer processes. It requires explicit modeling of the coupled electronic and vibrational dynamics, which is not a trivial task. One of the first attempts was done by Liang Shen, who considered total energy changes accompanying possible reactions of porphyrin, chlorin, and porphyrazine (abbreviated below as PS) with molecular oxygen at the B3LYP/6-31+G(d,p) level [107]:

PS(T_1_) + ^3^O_2_ → PS(S_0_) + ^1^O_2_ (1)

PS(T_1_) + ^3^O_2_ → PS(S_0_)**^⋅^**^+^ + O_2_**^−^** (2)

PS^−^ + ^3^O_2_ → PS + O_2_**^−^** (3)

It was postulated that the singlet oxygen can be generated according to reaction (1) in water and benzene by all studied porphyrinoids, whereas O_2_**^−^** can be obtained only in water according to reactions (2) and (3), whereas mechanism (3) can involve only porphyrazine as the photosensitizer.

More advanced calculations in which the dynamics of the PS to O_2_ energy transfer process are studied, are to the best of our knowledge, non-existent.

### 2.5. Quantitative Structure–Activity Relationship (QSAR) Studies

QSAR studies aiming at elucidation of relationships between structural and electronic properties of photosensitizers and their activity in PDT are scarce.

The oldest QSAR analyses link porphyrinoid activity with the octanol/water partition coefficient and basic structural parameters (length and shape of alkyl chains) [139,140]. Banfi and co-workers proposed predicting the photodynamic cytotoxicity of porphyrins (bearing variable numbers of aryl substituents in *meso*-positions) towards human colon adenocarcinoma cells [141]. Three molecular descriptors were selected by genetic algorithms as the most relevant to obtaining models with the highest predictive power. These were: (i) the autocorrelation descriptor GATS6v [142], which accounts for the correlation among atoms, weighted by the van der Waals volumes, with a distance of six bonds in the molecule; (ii) the topological descriptor PW3 [143], which is linked to molecular shape information; (iii) GETAWAY descriptor R4u+ [144], which accounts for the local structural aspects of the molecule.

Bettanin et al. also searched for the best descriptors of the photodynamic activity of porphyrin, phthalocyanine, and chlorin [94]. They proposed molecular volume, LUMO energy, oscillator strength, dipole moment, and free energy of solvation as the most relevant for the desired activity.

The experimental Q_x_ band energy of metallobacteriochlorins (M-BChl) correlates with the HOMO–LUMO energy gap determined at the BP/def2-TZVP level [145]; therefore, the latter can be used to predict the adsorption edges of the unknown derivatives of M-BChl.

One can see that the photodynamic activity of the studied set of porphyrins is mostly determined by such structural aspects as their sizes and shapes, and descriptors of their solubility in water (octanol/water partition coefficient, free energy of solvation, or dipole moment). 

## 3. Conclusions

1. As can be seen, TD-DFT is one of the most widely applied methods in these studies, due to its good balance between accuracy and computational cost. The fair agreement between theoretical and experiment spectra found for various porphyrinoids indicates that an a priori computation of some important photophysical properties may guide the design of new drugs that can be proposed as potential photosensitizers for photodynamic therapy.
2. Most studies advise the use of B3LYP or M06-2X functionals for geometry optimization and the CAM-B3LYP functional for predicting the UV–VIS spectra of porphyrinoids. The latter well handles charge-transfer states, which dominate the photochemistry of these macrocycles.
3. The inclusion of a solvent usually induces only minor geometry changes in the porphyrinoids. There are no significant differences in outcome among the implicit solvent models that are employed for the calculations.
4. The S_0_–T_1_ gap is generally well reproduced by the B3LYP or PBE0PBE functionals.
5. The spin-orbit couplings, computed at the B3LYP level, quantifying the singlet-to-triplet intersystem crossing, scale well with such experimental data as intersystem crossing yields and singlet oxygen quantum yields.
6. Regarding the interactions between the photosensitizer and molecular oxygen, the relevant calculations are still to be determined. The mechanism of the energy transfer between the photosensitizer and molecular oxygen is sought, as pathways involved in this process are essential to the design of effective PDT drugs. Studies similar to [146] are highly demanded.
7. QSAR analyses indicate that the structural parameters characterizing porphyrinoids’ shapes, volumes, and solubility levels are the most important factors determining their photodynamic activity. This is in line with the common observation that an important parameter for anticancer drugs is their solubility in aqueous solutions.

## Figures and Tables

**Table 1:** The mean errors (ME), the mean absolute errors (MAE), and absolute maximum errors (MAXE) determined by comparing theoretical (RPA approach, def2-SVP basis set) and experimental spectra of various porphyrinoids (porphyrin, octaethylporphyrin, Mg octaethylporphyrin, Zn octaethylporphyrin, tetraphenylporphyrin, Mg tetraphenylporphyrin, Zn tetraphenylporphyrin, tetrakis(*o*-aminophenyl)porphyrin, Zn tetrakis(4-carboxyphenyl)porphyrin, Zn [5,15-dipiridyl- 10,20-bis(pentafluorophenyl)] porphyrin, Zn [5,15-di(pyridylacetyl)-0,20-diphenyl] porphyrin, octabromotetraphenyl porphyrin) determined with different functionals—data taken from [83].

Functional	ME [eV]	MAE [eV]	MAXE [eV]
GGA and meta-GGA functionals			
PBE	−0.04	0.11	0.48
BP89	−0.04	0.11	0.47
BLYP	−0.05	0.10	0.46
TPSS	0.00	0.10	0.38
M06-L	0.05	0.11	0.26
Global hybrid functionals			
PBE0	0.15	0.15	0.25
B3P86	0.13	0.13	0.24
B3LYP	0.12	0.12	0.23
TPSS0	0.16	0.16	0.27
M06	0.07	0.10	0.17
BHLYP	0.14	0.14	0.22
M06-2X	0.15	0.15	0.23
Range separated hybrid functionals			
ωB97	−0.16	0.16	0.23
ωB97X	−0.08	0.09	0.17
LC-BLYP	−0.09	0.10	0.18
CAM-B3LYP	0.07	0.08	0.16

**Table 2:** Performances of the CAM-B3LYP functional when reproducing experimental Q and B bands of selected porphyrinoids.

System	Basis-Set	Q [nm]	B [nm]	Exp. [nm]	Ref.
H_2_P (free-base porphyrin)	6-31G	502	331	Q 514 B 372	[51]
Zn-P	6-31G	512	334	Q 569–532 B 396	[51]
Zn-P	6-31G(d)	517	335	Q 572 B 402	[86]
Zn-TPP	6-31G(d)	541	359	Q 586 B 419	[86]
5,10,15,20-tetrakis(4-hydroxyphenyl)-porphyrin (p-THPP)	6-31G**	Q_y_ 590 Q_x_ 539	n/d	Q_y_ 651, 594 Q_x_ 553, 516	[93]
Phtalocyanine (Pcs)	6-311G(d,p)	Q_y_ 624 Q_x_ 610	295	Q_y_ 654 Q_x_ 689 B 337	[94]
Zn-Pcs	6-311G(d,p)	612	296	Q 667 B 343	[94]
Zn-Pcs	6-31G(d)	607	313, 295	Q 670 B 370, 330	[86]

**Table 3:** Performances of the B3LYP functional when reproducing experimental Q and B bands of selected porphyrinoids.

System	Basis-Set	Q [nm]	B [nm]	Exp. [nm]	Ref.
H_2_P (free-base porphyrin)	6-31G	500	348	Q 514 B 372	[51]
Zn-P	6-31G	506	347	Q 569-532 B 396	[51]
Zn-P	6-31G(d)	507	349	Q 572 B 402	[86]
Zn-TPP	6-31G(d)	535	381	Q 586 B 419	[86]
*meso*-tetrakis(3-metoxy-4-hydroxyphenyl)-porphyrin	6-31G(d)	Q_y_ 599.01 Q_x_ 563.37	435.12; 431.77; 425.25; 423.87; 407.23	Q_y_ 651,594 Q_x_ 553, 516 B 421, 403	[89]
*meso*-tetra (hydroxyphenyl) porphycene (m-THPPo, temocene),	6-31G(d)	594, 571	n/d	656, 624, 583	[95]
5,10,15,20-tetrakis- (m-hydroxyphenyl)chlorin (m-THPC, Foscan)	6-311+G(d,p)	558, 523	n/d	650, 414	[95]
5,10,15,20-tetrakis(4-hydroxyphenyl)-porphyrin (p-THPP)	6-31G	Q_y_ 587 Q_x_ 544	n/d	Q_y_ 651, 594 Q_x_ 553, 516	[93]
p-THPP	6-31G**	Q_y_ 588 Q_x_ 552	n/d	Q_y_ 651, 594 Q_x_ 553, 516	[93]
p-THPP	6-31G+**	Q_y_ 585 Q_x_ 549	n/d	Q_y_ 651, 594 Q_x_ 553, 516	[93]
B-ring benzoporphyrin derivative B3B^−^	6-311+G(d,p)	Q_y_ 626 Q_x_ 556	442, 437, 409	Q_y_ 689, 628 Q_x_ 564, 502 B 432, 416	[88]
B-ring benzoporphyrin derivative B3B^0^	6-311+G(d,p)	Q_y_ 636 Q_x_ 588	474, 466, 412, 403	Q_y_ 688, 628 Q_x_ 576, 546 B 432	[88]
B-ring benzoporphyrin derivative B3B^2+^	6-311+G(d,p)	Q_y_ 668 Q_x_ 592	467, 460, 430, 419	Q_y_ 672,616 Q_x_ 592, 552 B 432	[88]
Phtalocyanine (Pcs)	6-311G(d,p)	Q_y_ 602 Q_x_ 597	328	Q_y_ 654 Q_x_ 689 B 337	[94]
Zn-Pcs	6-311G(d,p)	596	323	Q 667 B 343	[94]
Zn-Pcs	6-31G(d)	593	366, 338	Q 670 B 370, 330	[86]

## Data Availability

Not applicable.
